# Development of a simplified version of the smoking rationalization belief scale for Chinese male smokers

**DOI:** 10.3389/fpsyt.2023.1044929

**Published:** 2023-02-20

**Authors:** Lingyun Zhang, Hao Chen, Yimeng Mao, Shichen Zheng, Pinpin Zheng

**Affiliations:** ^1^School of Public Health, Fudan University, Shanghai, China; ^2^Key Laboratory of Public Health Safety, Ministry of Education, Fudan University, Shanghai, China; ^3^Department of Public Health Sciences, University of California, Davis, Davis, CA, United States

**Keywords:** smoke, smoking rationalization belief, simplified scale, China, male smokers

## Abstract

**Objective:**

The goal of this study was to simplify the smoking rationalization belief (SRB) scale among Chinese male smokers and provide a convenient measuring tool with good reliability and validity to promote the assessment and further intervention of SRB among smokers.

**Methods:**

Through purposive sampling, a questionnaire survey was conducted among adult male smokers in three districts in Shanghai, and 1,307 valid questionnaires were collected. Exploratory factor analysis was used to analyze the simplified scale, and Pearson correlation analysis, multiple linear regression, and Cronbach’s α were used to test the reliability and validity of the simplified scale.

**Results:**

The SRB scale was simplified from 26 items to 8 items and had good overall reliability (Cronbach’s α = 0.757). There was a strong correlation between the simplified scale and the original scale (*P* < 0.001, *r* = 0.911), and the scores of SRB measured by the two scales were both negatively associated with a willingness to quit smoking (*P* < 0.001), which reflected the practical effectiveness of the simplified version.

**Conclusion:**

The simplified version of the SRB scale showed good reliability and validity among Chinese smokers, which facilitates smoking cessation-related research and practice.

## Introduction

China is the largest tobacco producer and consumer globally (1). According to the 2018 China Adult Tobacco Survey Report, the total number of smokers among people aged 15 and over in China has exceeded 300 million and 50.5% of men were smokers (2). Smoking cessation is the most direct and effective way of reducing tobacco use and the disease burden caused by tobacco use (3). However, the willingness to quit smoking among Chinese smokers is generally low, and only 16.1% of smokers intend to quit smoking in the next 12 months, which represents an important barrier to efforts to decrease the smoking prevalence in China (2).

Previous studies have confirmed that perceived smoking hazards, self-efficacy in quitting smoking, and nicotine dependence were important factors that influence quitting intention among Chinese smokers (4). However, traditional intervention measures, such as educating smokers on the hazards of smoking, have had minimal effects on improving Chinese smoker’s willingness to quit smoking (5). Cognitive dissonance theory implies that when there is an inconsistency (dissonance) between attitudes or behaviors, the underlying tension could motivate an individual to change their attitude to provide greater consistency between thoughts and behaviors (6). Smoking rationalization belief (SRB) refer to the relevant attitudes that smokers use to rationalize their smoking behavior and maintain their smoking status. Some studies have shown that SRBs are common among smokers, as many smokers are sceptical about the harm of smoking. Chapman et al. found that 29.9% of smokers and recent quitters disagree that exposure to second-hand smoke can maintain widespread levels of lung cancer in Australia (7). Another study on Chinese smokers showed that some Chinese people considered tobacco to be an important part of the national tax revenue source, so smoking became almost a patriotic duty (8).

Smoking rationalization beliefs have both direct and indirect negative effects on the intention to quit smoking. For example, holding the belief that “smoking is worth it” is a strong predictor of decreased quitting intention. On the other hand, smokers with such beliefs are less likely to identify the hazards and diseases caused by smoking, thus further hindering their willingness to quit (7, 9–11). Such beliefs are affected by policy, culture and industries’ marketing. Identifying rationalization beliefs and constructing the related scale are necessary to developing further intervention to dispel the misunderstanding of smokers and promote quitting willingness. Several scales have been developed to measure common smoking rationalization beliefs, such as smoking functional beliefs measures and disengagement beliefs scales. Although validated, these measures were mostly developed in western countries and few studies focused on SRBs in Asian countries (12–14).

In 2016, a Chinese male smokers’ smoking rationalization belief scale was developed that used 26 items in six dimensions, including smoking functional beliefs, risk generalization beliefs, social acceptability beliefs, safe smoking beliefs, self-exempting beliefs, and quitting is harmful beliefs (11). Such beliefs were widely spread among smokers. In a survey among Chinese smokers in three cities, more than half of smokers agreed with each of the top 10 SRBs (15). This scale comprehensively reflects smokers’ beliefs regarding smoking rationalization and facilitates targeted tobacco-control health education. It also shows good reliability and validity and has been applied in tobacco control research and practice. For example, the scale was applied to a examine the association between rationalization belief and the intention to quit where the result indicated that “believing that smoking was socially acceptable” was the strongest predictor of quitting intention (16). However, the scale’s use of 26 items extends the necessary time for the measurement and limits further application. Thus, it is important to simplify the scale appropriately, which would facilitate the popularization and use of the scale.

In fact, many studies have simplified the scale according to their needs. For example, Mona et al. simplified the state anxiety scale (SAI) with 20 items by factor analysis and extracted six items to form a simplified version of the SAI, which has been widely used to measure the anxiety of patients (17). Furthermore, the simplified symptom checklist-90 (SCL-90), which is based on the machine learning model, has been applied to intelligent psychological and physical examination services in many health centers (18). Therefore, simplifying the scale can avoid the negative influence of a lengthy scale on survey quality and reduce the difficulty and the cost of investigation to ensure the respondents’ compliance and increase the representativeness and authenticity of the survey (19).

The simplification of the scale is mainly meant to screen items with solid representativeness, sound discrimination, and good internal consistency based on the original scale. This study aims to simplify the smoking rationalization belief scale and analyze the reliability and validity of the simplified scale (20).

## Materials and methods

### Participants and procedure

Purposive sampling was used to improve the representativeness and three districts were selected in Shanghai—including Changning District (urban area), Minhang District (urban-suburb binding area), and Qingpu District (suburb area)—in 2019. According to the regional GDP ranking of 16 districts in Shanghai in 2018, Minhang, Changning and Qingpu ranked fourth, eighth and twelfth respectively, representing the different economic development levels (21).

In each selected district, we worked with the Center for Disease Control and Prevention (CDC) of the local district to choose workplaces for the study that represented different occupational populations. We used random sampling methods to select participants from the list of all smokers provided by workplace contactors. Officers and clerks in government/institutions and corporations, professionals, businessmen, service employees, farmers and manual workers were sampled from their workplaces, and students were sampled from universities/colleges. The number of smokers recruited in different occupations reflected the distribution of occupational categories according to the 2018 Chinese Statistical Yearbook (22). In each workplace, we obtained the list of smokers as provided by workplace contactors. If a workplace liaison group agreed to participate, then all smokers from that workplace were invited to participate until the target number was reached. Such a sampling strategy is helpful in involving smokers with different occupations from a variety of workplaces. To balance the proportions of participants who were working with those who had retired (23), we supplemented workplace-based sampling with community sampling to recruit retirees. The inclusion criteria were as follows: (1) Adult males, (2) smokers who had smoked at least 100 cigarettes over their life and still smoked at the time of inclusion (24), (3) smokers with no history of neurological diseases or psychosis, and (4) smokers who were able to complete the questionnaire independently. Exclusion criteria included: (1) Having severe mental illness and cognitive impairment; (2) refusing to participate in this study. The sample size of the each subsample should be 5–10 times of the number of the original scale items (25). Assuming that the invalid questionnaire rate was 20%, we increased the single subsample size to 500, so the total size of the two subsamples was 1,000. Participants who consented to participate in the study were invited to complete an anonymous self-administered questionnaire consisting of the 26-item rationalization scale and other questions, such as smoking-related behavior, nicotine independence, intention to quit and demographic information. Trained research assistants were present during the survey. They distributed printed questionnaires to the respondents, and let the respondents finish the questionnaire by self-filling. They could also provide help if the respondents have any questions. A valid questionnaire in this study means that main questions (including demographic information, smoking status and questions of SRB) were answered completely without obvious logical error. A quality control item with a required answer was included to avoid the return of invalid questionnaires. Moreover, questionnaires with the same answer being provided to for all questions were also excluded. A total of 1,307 valid questionnaires from 1,387 male smokers were collected with a valid completion rate of 94.2%. The Ethics Committee for Medical Research at the School of Public Health, Fudan University, approved this study (IRB00002408 and FWA00002399).

### Measurements

#### SRBs

The original version of the smoking rationalization belief scale was used to evaluate the SRBs of the participants. The scale includes 26 items divided into six dimensions: smoking functional beliefs, risk generalization beliefs, social acceptability beliefs, safe smoking beliefs, self-exempting beliefs, and quitting is harmful beliefs (Table 1). For each question, “strongly agree,” “agree,” “neutral,” “disagree,” and “strongly disagree” were scored as 1, 2, 3, 4, and 5 points, respectively, on a 5-point Likert scale. The higher the score was, the lower the SRBs. The total Cronbach’s α of the scale was 0.939 which showed good reliability. The scale also had acceptable validity including convergent validity, nomological validity and discriminant validity (11).

**TABLE 1:** The original version of the smoking rationalization belief scale.

Factor	Items
F1: smoking functional beliefs	S14 “Smoking can eliminate fatigue and be refreshing.”
	S15 “Smoking is good for inspiration and active thinking.”
	S17 “Smoking can reduce interpersonal distance and make social interaction easier.”
	S16 “Smoking is a good way to kill time.”
	S13 “Smoking can relieve tension and stress.”
F2: Risk generalization beliefs	S6 “Air pollution, food safety, and life stress are much more dangerous to health than smoking.”
	S8 “If smoking was so bad for health, the government would have banned tobacco sales.”
	S3 “A lot of non-smokers also get lung cancer.”
F3: Social acceptability beliefs	S19 “Many famous people smoke, so it is normal to smoke.”
	S20 “Smoking is pretty normal for men.”
	S18 “There are so many smokers in society that it’s hard for one to be different.”
	S26 “Lots of doctors smoke, so it’s unconvincing for them to persuade me to quit.”
	S25 “I will consider quitting smoking only if the government closes the tobacco factory.”
	S21 “Smoking is a part of my lifestyle that others can’t interfere with.”
F4: Safe smoking beliefs	S11 “Low-tar cigarettes can reduce the harms of smoking/is less harmful.”
	S9 “If you don’t inhale the smoke into the lungs, the harm is minimized.”
	S10 “People like me who do not smoke many cigarettes are not at risk of smoking health problems.”
	S12 “It’s safe to smoke high-quality cigarettes.”
F5: Self-exempting beliefs	S2 “I have not experienced any harm to my health.”
	S4 “I think I may have genes which protect me from the harms of smoking.”
	S5 “Smoking is not always bad for you because many smokers live long lives while many non-smokers don’t.”
	S1 “There is still insufficient medical evidence to prove that smoking is harmful.”
	S7 “The fact that I can still smoke means my health status is not bad.”
F6: Quitting is harmful beliefs	S22 “If you have smoked for a long time, the body has adapted to smoking and reached a balance, so quitting will lead to illness.”
	S24 “After quitting smoking, I will gain weight, which is also harmful to my health.”
	S23 “If you try to quit and fail, you will smoke even more than before, so it is better not to quit.”

### Knowledge about smoking and health

Knowledge of smoking-related diseases was measured by four “yes/no” questions regarding the following: Whether smoking causes heart disease, emphysema, gastric carcinoma and impotence (26). Choosing “yes” gets 1 point in each option while choosing “no” gets 0 point. The sum of the scores of all questions ranged from zero to four.

### Tobacco uses

Tobacco use included four aspects: Smoking history (i.e., age of smoking initiation, years of smoking, and the number of cigarettes smoked per day), quitting attempts and smoking cessation experiences (times and duration) and nicotine dependence.

The Fagerstrom Test for Nicotine Dependence (FTND) was applied to evaluate nicotine dependence. The score of 0∼4 represents low nicotine dependence, the score of five represents moderate nicotine dependence, and the score of ≥6 is considered as high nicotine dependence (27).

According to the theory of stage change, quitting attempts were divided into several stages, including precontemplation (not having considered quitting smoking within 6 months), contemplation (preparing to quit smoking within 6 months), preparation (planning to quit smoking within 1 month), action (starting to quit, or quitting for less than half a year) and maintenance (having quit smoking more than half a year prior).

### Demographic information

Demographic information, including age, education level, occupation, marital status, income, and self-reported health status, was also collected in the questionnaire.

### Statistical analysis

The non-dimension reduction method was used to simplify the scale; that is, the simplified SRB scale still retains the dimensions of the original scale. After reviewing previous literature on scale simplification (28–31), the data analysis was mainly performed in three stages: (1) We randomly divided the data into two groups according to a random number generator, using the Bartlett spherical test and Kaiser Meyer-Olkin (KMO) test to evaluate the applicability of factor analysis (2). We used factor analysis among the two subsamples. Principal component analysis was applied to extract dimensions, and then rotations were made using the maximum variance method. According to the idea that the characteristic value was greater than 1, six dimensions were extracted. Then, after principal component analysis, we used the following principles as items’ exclusion criteria: (1) Having multiple factors loaded in each dimension (≥2), with a minimum factor load value of 0.30, that is, the factor load value of one item in multiple dimensions should be greater than 0.30; (2) Having no main factor load in each dimension, with a minimum value of the main factor load defined as 0.70; (3) Having a CITC value of less than 0.50. In addition, if there were no remaining items in a dimension after deleting items, the item with the largest factor load in the dimension was retained to ensure that there was at least one item retained in each dimension (17, 32, 33). Therefore, the structure of the simplified SRB scale was cross verified (11, 17, 34). This step, in addition to shortening the scale and balancing the number of items across factors, may have enhanced the stability of the scale (32) (3). Using the whole sample (1,307 records), we tested the reliability and validity of the simplified SRB scale. First, we used Pearson correlation analysis to evaluate the correlation between rationalization belief scores as measured by the original and simplified scales. Additionally, we conducted multiple linear regression and multivariate logistic regression analysis to analyze and compare factors that affect SRBs scores as measured by the original and simplified scales. SPSS 21.0 (SPSS, Chicago, IL, USA) was applied to analyze the data.

## Results

### Sample characteristics

The average age of the respondents was 41.58 ± 14.41 years old, among which young people under the age of 30 accounted for the largest proportion (26.4%). The number of professional technicians accounted for 27.5% of all respondents. Over 80% were married, and the average level of income per month was 2000–3999 Yuan (42.9%). Most of the respondents (50.7%) rated their health as fair, and 43.5% rated their health as good (Table 2).

**TABLE 2:** Demographic characteristics of respondents.

Demographics	Total (*N* = 1,307)	Subsample I (*N* = 651)	Subsample II (*N* = 656)	*P*-value
Age (years)				0.082
≤30	345 (26.4)	196 (56.8)	149 (43.2)	
31–40	312 (23.9)	148 (47.4)	164 (52.6)	
41–50	333 (25.5)	149 (44.7)	184 (55.3)	
51–60	170 (13.0)	85 (50.0)	85 (50.0)	
≥61	147 (11.2)	73 (49.7)	74 (50.3)	
Education				0.457
Primary school or lower	75 (5.7)	35 (46.7)	40 (53.3)	
Junior school	308 (23.6)	143 (46.4)	165 (53.6)	
High school/Technical school	350 (26.8)	175 (50.0)	175 (50.0)	
Bachelor’s degree	230 (17.6)	125 (54.3)	105 (45.7)	
Master’s degree or higher	344 (26.3)	173 (50.3)	171 (49.7)	
Occupation^a^				0.963
Person in charge/Manager	23 (1.8)	11 (47.8)	12 (52.2)	
Professional technician	359 (27.5)	172 (47.9)	187 (52.1)	
Clerk	111 (8.5)	60 (54.1)	51 (45.9)	
Business people	166 (12.7)	83 (50.0)	83 (50.0)	
Service personal	149 (11.4)	75 (50.3)	74 (49.7)	
Farmer	93 97.1)	46 (49.5)	47 (50.5)	
Manual worker	227 (17.4	116 (51.1)	111 (48.9)	
Student	81 (6.2)	43 (53.1)	38 (46.9)	
Retiree	98 (7.5)	45 (45.9)	53 (54.1)	
Married status				0.107
Married	1,054 (80.6)	513 (48.7)	541 (51.3)	
Not married	253 (19.4)	138 (54.5)	115 (45.5)	
Monthly income (Yuan^b^)				0.327
<2,000	192 (14.7)	94 (49.0)	98 (51.0)	
2,000–3,999	561 (42.9)	283 (50.4)	278 (49.6)	
4,000–5,999	372 (28.5)	194 (52.2)	178 (47.8)	
≥6,000	182 (3.9)	80 (44.0)	102 (56.0)	
Self-assessment of health				0.423
Poor	77 (6.0)	38 (49.4)	39 (50.6)	
Fair	662 (50.7)	343 (51.8)	319 (48.2)	
Good	538 (43.5)	270 (47.5)	298 (52.5)	–

^a^According to occupational classification code of the people’s Republic of China (2015 version).

^b^1 dollar = 6.36 Yuan (2022).

### Simplification of the SRB scale

The 1,307 samples were randomly divided into two subsamples of 651 and 656 records respectively through random number table. There was no significant difference between the demographic characteristics of the two subsamples (Table 2). The Bartlett test and KMO test were independently conducted on two subsamples. The results showed that the *P*-value of the Bartlett test of subsample I was less than 0.001, and the KMO value was 0.949. The Bartlett test *p*-value of subsample II was less than 0.001, and the KMO value was 0.949. Therefore, the KMO values of subsamples I and II both met the conditions of factor analysis.

According to the above rules of deleting and retaining items, and after factor analysis of subsample I, three items (S13, S14, and S15) were included in Dimension 1 (smoking functional beliefs). For Dimension 2 (self-exempting), Dimension 3 (social acceptability), Dimension 4 (safe smoking), Dimension 5 (quitting is harmful), and Dimension 6 (risk generalization), there was only one item retained in each dimension, named S1, S25, S11, S23, and S6, respectively (Table 3).

**TABLE 3:** Factor analysis of subsample I.

Items	Factor loading						CITC value	Reason for deletion	Reason for reservation
	Dimension 1	Dimension 2	Dimension 3	Dimension 4	Dimension 5	Dimension 6			
S1	0.051	0.702	0.035	0.053	0.151	0.056	0.420		D
S2	0.226	0.695	0.043	0.158	0.155	0.199	0.586	B	
S3	0.252	0.345	0.008	0.053	0.151	0.530	0.485	A, B	
S4	0.116	0.682	0.170	0.195	0.192	-0.004	0.553	B	
S5	0.125	0.638	0.226	0.172	0.255	0.255	0.659	B	
S6	0.135	0.142	0.144	0.135	0.219	0.697	0.509		D
S7	0.102	0.465	0.155	0.261	0.459	0.202	0.635	A, B	
S8	-0.004	0.118	0.471	0.125	-0.035	0.531	0.428	A, B	
S9	0.113	0.127	0.175	0.655	0.101	0.217	0.511	B	
S10	0.118	0.222	0.206	0.650	0.230	0.072	0.571	B	
S11	0.206	0.081	0.000	0.726	0.206	0.055	0.467		
S12	0.202	0.274	0.058	0.648	0.280	-0.015	0.557	B	
S13	0.755	0.048	0.109	0.126	0.010	0.264	0.502		
S14	0.763	0.147	0.117	0.130	-0.012	0.114	0.503		
S15	0.735	0.167	0.132	0.140	0.136	0.043	0.542		
S16	0.572	0.139	0.244	0.039	0.300	-0.019	0.515	A, B	
S17	0.707	-0.004	0.145	0.124	0.172	0.104	0.488	C	
S18	0.369	0.237	0.623	0.219	0.060	-0.086	0.590	A, B	
S19	0.405	0.276	0.627	0.176	0.157	-0.020	0.669	A, B	
S20	0.433	0.135	0.522	0.245	0.146	0.120	0.637	A, B	
S21	0.452	0.159	0.386	0.171	0.306	0.137	0.634	A, B	
S22	0.134	0.365	0.054	0.185	0.638	0.154	0.585	A, B	
S23	0.147	0.241	0.220	0.165	0.652	0.218	0.628		D
S24	0.088	0.208	0.216	0.310	0.596	0.084	0.576	A, B	
S25	0.107	0.114	0.721	-0.022	0.206	0.181	0.509		
S26	0.201	0.153	0.579	0.109	0.287	0.328	0.638	A, B	

Deletion criteria:

A: There were multiple factor loads, and the minimum factor load was 0.30.

B: There was no main factor load, and the minimum value of main factor load was 0.70.

C: The CITC value was less than 0.50.

Reservation criteria:

D: Factor retained to ensure that there was at least one item in each dimension without dimension reduction.

According to the above principles, factor analysis was applied to subsample II to delete and retain items. As a result, three items (S17, S18, and S19) were retained in Dimension 1; Dimension 2, Dimension 3, Dimension 4, Dimension 5, and Dimension 6 all retained one item, as S1, S25, S11, S24, and S3, respectively (Table 4).

**TABLE 4:** Factor analysis of subsample II.

Items	Factor loading						CITC value	Reason for deletion	Reasons for reservation
	Dimension 1	Dimension 2	Dimension 3	Dimension 4	Dimension 5	Dimension 6			
S1	0.051	0.760	-0.092	0.117	0.115	0.066	0.414		
S2	0.152	0.634	0.117	0.194	0.265	0.160	0.604	B	
S3	0.115	0.162	0.114	0.156	0.214	0.678	0.457		D
S4	0.109	0.523	0.223	0.302	0.289	-0.075	0.569	A, B	
S5	0.188	0.660	0.139	0.198	0.232	0.170	0.631	B	
S6	0.232	0.245	0.164	0.145	0.052	0.640	0.497	B	
S7	0.083	0.323	0.210	0.300	0.512	0.200	0.610	A, B	
S8	0.067	0.398	0.420	-0.127	0.037	0.285	0.372	A, B	
S9	0.164	0.139	0.048	0.677	0.226	0.129	0.542	B	
S10	0.182	0.334	0.168	0.614	0.225	0.085	0.648	B	
S11	0.181	0.145	0.078	0.681	0.214	0.084	0.548		D
S12	0.218	0.331	0.097	0.551	0.293	0.067	0.632	A, B	
S13	0.716	0.009	0.089	0.108	0.153	0.244	0.524		
S14	0.772	0.074	0.106	0.126	0.157	0.057	0.551		
S15	0.705	0.206	0.189	0.134	0.169	-0.068	0.588		
S16	0.563	0.178	0.151	0.245	0.172	-0.033	0.547	B	
S17	0.680	0.024	0.110	0.062	0.016	0.260	0.456	B	
S18	0.362	0.187	0.503	0.387	-0.104	0.043	0.557	A, B	
S19	0.423	0.321	0.503	0.326	0.013	-0.041	0.649	A, B	
S20	0.495	0.169	0.417	0.325	0.040	0.005	0.601	A, B	
S21	0.455	0.157	0.262	0.353	0.233	0.153	0.641	A, B	
S22	0.296	0.258	-0.034	0.260	0.667	0.055	0.601	B	
S23	0.263	0.166	0.198	0.148	0.643	0.133	0.592	B	
S24	0.105	0.211	0.252	0.150	0.682	0.057	0.553		D
S25	0.214	0.068	0.716	0.026	0.249	0.134	0.513		
S26	0.234	0.010	0.638	0.183	0.296	0.129	0.551	B	

Deletion criteria:

A: There were multiple factor loads, and the minimum factor load was 0.30.

B: There was no main factor load, and the minimum value of main factor load was 0.70.

C: The CITC value was less than 0.50.

Reservation criteria:

D: Factor retained to ensure that there was at least one item in each dimension without dimension reduction.

The above results show that after factor analysis of two independent subsamples, the items retained by Dimension 1, Dimension 2, Dimension 3, and Dimension 4 were the same, but the items retained by Dimension 5 and Dimension 6 were different. For subsample II, the CITC value of Item S3 retained by Dimension 6 was 0.457, which is less than 0.5, while the CITC value of Item S6 retained by Dimension 6 in subsample I was 0.509, which is greater than 0.5. According to the rule that the CITC value should be greater than 0.5 to ensure better reliability, Dimension 6 would keep Item S6.

For Dimension 5, the CITC values of the items retained by the two subsamples were both greater than 0.5, which could not be directly chosen by the CITC rule. Therefore, we compared the reliability of the two simplified scales with different dimension 5 through the Cronbach’s α test. Scale I consisted of eight items: S1, S6, S11, S13, S14, S15, S23, and S25, with Cronbach’s α of 0.742. Scale II consisted of eight items: S1, S6, S11, S13, S14, S15, S24, and S25, and the Cronbach’s α was 0.754. Therefore, the reliability of scale II was better than that of scale I, and the CITC value of S23 was smaller than that of S24. Based on the above index results, S24 was selected as the item of Dimension 5. Therefore, the final items of the simplified SRB scale included S1, S6, S11, S13, S14, S15, S24, and S25, which accounts for 89.657% of the total variance.

### Reliability of the simplified SRB scale

Internal consistency reliability was assessed by calculating Cronbach’s α. The overall Cronbach’s α of the 8-item simplified was 0.757, which is greater than 0.7 and indicated a high internal consistency reliability for the short form of the SRB scale.

### Validity of the simplified SRB scale

#### Criterion validity

We calculated each dimension’s SRB scores for both the original and simplified smoking rationalization scales. The results of Pearson correlation analysis show that the scores of all dimensions of the two scales were significantly and strongly correlated (correlation coefficient *r* > 0.7, *P* < 0.001), which showed a good correlation between the original scale and the simplified scale (Table 5).

**TABLE 5:** Correlation analysis of dimensions between the original scale and the simplified scale.

Dimension	Original scale	Simplified scale	Correlation coefficient *r*	*P*-value
Smoking functional beliefs	13, 14, 15, 16, 17, 20, 21	13, 14, 15	0.894	<0.001
Self-exempting beliefs	1, 2, 4, 5, 7	1	0.714	<0.001
Social acceptability beliefs	18, 19, 25, 26	25	0.773	<0.001
Safe smoking beliefs	9, 10, 11, 12	11	0.777	<0.001
Quitting is harmful beliefs	22, 23, 24	24	0.807	<0.001
Risk generalization beliefs	3, 6, 8	6	0.846	<0.001

#### Content validity

The scores of each dimension of the simplified SRBs scale were significantly associated with the total score, and the correlation coefficients between the scores of each dimension were also statistically significant (Table 6).

**TABLE 6:** Correlation between each dimension and total score of simplified smoking rationalization belief scale.

	Smoking functional beliefs	Self-exempting beliefs	Risk generalization beliefs	Safe smoking beliefs	Social acceptability beliefs	Quitting is harmful beliefs	Total score
Smoking functional beliefs	1.000						
Self-exempting beliefs	0.103**	1.000					
Risk generalization beliefs	0.238**	0.175**	1.000				
Safe smoking beliefs	0.242**	0.166**	0.216**	1.000			
Social acceptability beliefs	0.268**	0.118**	0.212**	0.122**	1.000		
Quitting is harmful beliefs	0.242**	0.252**	0.216**	0.271**	0.276**	1.000	
Total score	0.615**	0.3845**	0.452**	0.440**	0.467**	0.498**	1.000

**P < 0.01.

#### Construct validity

By controlling for confounding factors (demographic factors, nicotine addiction and self-assessment of health), the result of logistic analysis showed that the score of the simplified version of the SRB scale was a significant predictor of smoking cessation attempts (OR: 0.712, 95% CI: 0.573–0.886, *P* < 0.001). The score of the original scale also predicted smoking cessation attempts (OR: 0.698, 95% CI: 0.562–0.868, *P* < 0.05).

Using the SRB scores as measured by the original scale and the simplified scale as dependent variables, the results of multiple linear regression showed that the factors affecting the SRB scores as measured by the two scales were nearly the same, and they mainly included age, nicotine dependence and willingness to quit smoking (*P* < 0.001). A higher SRB score was associated with older age, higher nicotine dependence and a lower willingness to quit smoking (Table 7).

**TABLE 7:** Analysis of factors affecting SRB scores as measured by the original and simplified scale.

Influencing factor	Original scale				Simplified scale			
	β	SE	*t*-value	*P*-value	β	SE	*t*-value	*P*-value
Age	0.192	0.033	5.760	<0.001	0.402	0.114	3.517	<0.001
Education	0.701	0.359	1.955	0.051	0.175	0.114	1.533	0.126
Income per month	-0.713	0.471	-1.512	0.131	-0.197	0.150	-1.312	0.190
Marital status	2.342	1.131	2.070	0.039	0.757	0.352	2.149	0.032
Self-assessment of health	0.702	0.513	1.369	0.171	0.132	0.164	0.807	0.420
Nicotine dependence	3.043	0.468	6.496	<0.001	0.913	0.150	6.105	<0.001
Smoking and health knowledge score	-0.758	0.347	-2.182	0.029	-0.126	0.111	-1.135	0.257
Willingness to quit smoking	-4.296	0.457	-9.401	<0.001	-1.210	0.146	-8.307	<0.001

## Discussion

This study used data from 1,307 male smokers’ smoking questionnaires to simplify the SRB scale from 26 to 8 items. After verification, the simplified SRB scale also showed good reliability and validity. The simplified version presented a feasible and convenient method for evaluation and is expected to increase the respondence and quality of the survey, which in turn leads to a wider application of related research and intervention practice.

Methods of simplifying the scale include clustering dimension reduction and non-dimensional reduction methods. Clustering dimension reduction reclassifies the dimensions with good consistency into new dimensions and then conducts confirmatory factor analysis (35). If the dimensions of the original scale were retained, then the items with factor loadings that meet the requirements in each dimension were recombined into a new scale, and the reliability and validity of the scale were verified. This study adopts non-dimensional reduction methods to simplify the scale; that is, the simplified SRB scale still retains six dimensions of the original scale. The overall Cronbach’s α of the original scale was as good as 0.939. While the simplified scale has been reduced to 8 items, the Cronbach’s α was still greater than 0.7 (0.757), which is in the recommended interval of 0.7–0.9 (36, 37). Exploratory factor analysis was used to cross-verify the simplified version of the scale obtained from the use of two subsamples. Except for the items retained by Dimension 5, which need further reliability tests, the items retained in other dimensions were consistent and reliable. Items S23 and S24, which were reserved by Dimension 5 in the two subsamples, did not meet the requirements of the main load, but they were both reserved based on the rule of reserved dimension. Reliability analysis showed that the Cronbach’s α of the two-scale combinations were similar, but that the scale with a higher Cronbach’s α contained S24; therefore, S24 was retained. Therefore, the internal consistency and credibility of the scale were ensured. Therefore, the selection of items adopts the method of comprehensive evaluation selection from previous research (38, 39), which is more scientific and rigorous in ensuring the optimal simplified scheme.

According to the results of criterion validity, the correlation analysis between the original scale and the simplified scale showed that the correlation coefficient of the total score of each dimension was 0.921 (*P* < 0.001), and the corresponding correlation coefficient of each dimension was also greater than 0.7, thus showing a strong correlation (40, 41).

The content validity result confirmed that the lower scores of the scale in both the simplified version and original version were significant predictors of smoking cessation attempts; this agrees with the results of Yong et al. who found that “smoking improves life quality” (smoking functional belief) was negatively correlated with quitting smoking attempts (42).

The determinants of the SRB scores of the two versions of the scale were also similar, which reflected the effectiveness of the simplified version of the scale. Among these factors, age is an important factor influencing SRB scores: the older the age is, the higher the SRB score. This was consistent with the results of Wellman et al. who found that smokers who discounted the importance of long-term health concerns were older than smokers with higher levels of risk perception (43). Smokers with high nicotine dependence held higher rationalization beliefs, which can be explained by the findings of Shaik et al. (44) that the higher the nicotine dependence is, the greater the chronicity of cigarette smoking, which will lead to significant cognitive dysfunction through atherosclerotic and haemodynamic processes (45–48). In addition, the willingness to quit smoking has a negative effect on SRBs, which could be due to nicotine dependence being negatively related to quitting intentions, with this relationship being mediated by perceived behavioral control (PBC) over smoking cessation (49). Smokers who know that smoking causes diseases (smoking is not reasonable) were more willing to quit smoking (50).

Based on these findings, elderly individuals, especially those who have been highly addicted to nicotine, should be the target population in attempts to dispel SRBs. Furthermore, according to the average score of each dimension, we can choose dimensions with relatively high scores as key educational targets to reduce smokers’ misconceptions.

This study also has some limitations. First, the scale was only developed for male smokers in China and not tested among female smokers. Regarding the fact that female smoking prevalence has been on the rise in recent years (3), future study should also focus on the SRBs among female smokers which might be quite different from beliefs of male smokers. In addition, SRBs among smokers in different countries were also different, so this scale, which was designed for Chinese smokers, should be modified before being applied in other countries. Last, the original version of the scale was developed several years ago, so the scale did not include contents of beliefs related to new products (such as e-cigarettes or heated cigarettes), which need further research.

To summarize, we developed a parsimonious, validated and simple rationalization scale for male smokers in China. This simplified scale could reduce the burden on the respondents and the investigators, and increase the authenticity of the data. Therefore, as a convenient measuring tool, it could advance the future research on rationalization belief in smoking cessation and inform intervention strategies to dispel SRBs widely held among male smokers in China. It also provides insights and references for SRBs research in other countries. SRBs were formulated and influenced by the tobacco control policy, social norms and culture and reflected different background characteristics. Future studies should focus on exploring various dimensions of SRBs under different context, so as to formulate targeted strategies to improve smokers’ willingness to quit smoking.

## Data availability statement

The raw data supporting the conclusions of this article will be made available by the authors, without undue reservation.

## Ethics statement

The studies involving human participants were reviewed and approved by the Ethics Committee of the School of Public Health, Fudan University. The patients/participants provided their written informed consent to participate in this study.

## Author contributions

PZ was the director of the entire study. LZ reviewed the scientific literature, designed the methodology, analyzed the data, and described the results. YM collected the data. HC and SZ coordinated improvements to the study design and redaction manuscript. All authors contributed to the article and approved the submitted version.
